# The in vivo and in vitro protein quality of three hemp protein sources

**DOI:** 10.1002/fsn3.3652

**Published:** 2023-09-10

**Authors:** Matthew G. Nosworthy, Adam Franczyk, Jason Neufeld, James D. House

**Affiliations:** ^1^ Department of Food and Human Nutritional Sciences University of Manitoba Winnipeg Manitoba Canada; ^2^ Richardson Centre for Food Technology and Research Winnipeg Manitoba Canada; ^3^ Canadian Centre for Agri‐Food Research in Health and Medicine Winnipeg Manitoba Canada; ^4^ Department of Animal Science University of Manitoba Winnipeg Manitoba Canada; ^5^ Present address: Agriculture and Agri‐Food Canada Guelph Research & Development Centre Guelph Ontario Canada

**Keywords:** hemp, hemp hearts, protein concentrate, protein digestibility corrected amino acid score, protein efficiency ratio, protein isolate

## Abstract

In this work, the protein quality of defatted hemp hearts and protein‐enriched hemp fractions was determined. Protein quality was assessed using a rodent bioassay to evaluate growth and protein digestibility, while amino acid composition was determined via HPLC. A method for determining in vitro protein digestibility was compared to in vivo methodology and used to generate an in vitro protein quality score. The true protein digestibility of hemp protein 2, a hemp protein concentrate, was significantly lower than that of either defatted hemp hearts or hemp protein 1, a hemp protein concentrate (*p* < .05). While there was no relationship between the in vivo and in vitro measurements of protein digestibility (*R* ^2^ = .293, *p* = .459), there was a significant correlation between the protein digestibility‐corrected amino acid score (PDCAAS) determined in vivo and in vitro PDCAAS (*R* ^2^ = .989, *p* = .005). The protein efficiency ratio of hemp protein 1 was significantly lower than that of either defatted hemp hearts or hemp protein 2 (*p <* .05). These data highlight the nutritional capacity of hemp protein sources while also demonstrating the relationship between in vivo and in vitro methods for determining protein quality.

## Graphical abstract

This study assessed the protein quality of defatted hemp hearts as well as protein‐enriched hemp products using in vivo and in vitro methods. While PDCAAS was similar across all samples, PER was lower for hemp protein 1 compared to hemp protein 2 or defatted hemp hearts. These data highlight the nutritional capacity of hemp protein sources.

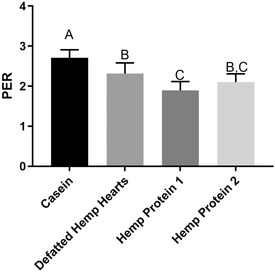

## 1 INTRODUCTION

The industrial production of hemp (*Cannabis Sativa* L.) was banned in many countries for the majority of the 20th century due to the presence of the psychoactive compound Δ‐9 tetrahydrocannabinol (THC) in the tissues of the plant. In 1998, Canada lifted this ban, permitting the breeding of hemp varieties containing low THC content, commonly less than 0.3% (Canada, 1998). Nutritionally, the oil content of hemp seed is between 33% and 35% (Callaway, 2004) and, after extraction, has been marketed as a dietary oil supplement. The protein content of hemp seed has been found to be approximately 25%, significantly higher than that of cereal‐based protein sources. Dehulled hemp seed, otherwise known as hemp hearts, has a higher fat and protein content than the whole seed, approximately 47% and 36%, respectively (House et al., 2010). The protein quality of hemp has also been demonstrated to be comparable to that of other plant protein sources (House et al., 2010).

The regulations governing the determination of protein quality differ depending upon the regulating body, with Canada relying on the protein efficiency ratio (PER) (HealthCanada, 1981) and the United States, as well as countries throughout Europe, relying on the protein digestibility corrected amino acid score (PDCAAS) (World Health Organization, 1991). PER, a rat bioassay, is a measurement of weight gain per quantity of protein consumed (HealthCanada, 1981). PDCAAS, another rat bioassay, compares the amino acid composition of a protein source to a reference requirement pattern, with values less than 1.0 indicating a limiting amino acid (World Health Organization, 1991). This amino acid score is then used in conjunction with the protein digestibility, determined via rodent bioassay, to generate a protein quality value.

The digestibility of plant proteins is lower than that of animal proteins due to the presence of anti‐nutritional factors such as trypsin inhibitors (Gupta, 1987; Oomah et al., 2011), tannins (Hahn & Rooney, 1986), and hemagglutinins (Bhatty & Christison, 1984). A study investigating these anti‐nutritional factors in hemp also detected the presence of tannins, phytic acid, saponins, and trypsin inhibitors in hemp flours derived from Italian and French varieties of *Cannibis sativa* (Russo & Reggiani, 2013). The presence of these compounds can reduce the in vivo digestibility of hemp protein; however, it has been previously demonstrated in other plants that processing reduces their presence or activity (Alonso et al., 2001; Marquardt et al., 1974; Roy et al., 2010).

Hemp products currently available include concentrates and isolates of hemp seed protein. Enriched protein fractions and protein concentrates are typically produced through air classification (Reichert, 1982), a mechanical process that selects for protein‐containing fractions by weight, whereas protein isolates are generated through a wet chemical method relying on protein solubility (Boye et al., 2010; Lam et al., 2018). These processes, either mechanical or chemical, increase protein content by removing non‐protein components; however, they may also modify factors influencing overall protein quality. While there has been some work performed on the protein quality of pulse protein concentrates and isolates (Nosworthy, Tulbek, & House, 2017), information regarding the quality of high‐protein hemp flours is lacking.

For that reason, this study was conducted to determine the PER and PDCAAS of two hemp protein concentrates as well as those of defatted hemp hearts. Additionally, the in vitro PDCAAS and a recently proposed method for determining protein quality, the digestible indispensable amino acid score (DIAAS) (World Health Organization, 2011), were also calculated for comparison with PDCAAS values.

## 2 MATERIALS AND METHODS

All procedures were approved by the Institutional Animal Care Committee in accordance with the guidelines of the Canadian Council on Animal Care (Protocol Number F2012‐035).

### 2.1 Chemicals

All chemicals and reagents were purchased from Sigma.

### 2.2 Sample procurement

Two samples of hemp protein concentrates (1 and 2) and one sample of dehulled hemp seeds (hemp hearts) were secured from local suppliers (Manitoba Harvest Hemp Foods; Hemp Oil Canada). Samples were secured from production lots that were manufactured in 2018.

### 2.3 Analytical procedures

For all samples, percent crude protein (CP; N × 6.25) was determined through the use of a LECO CNS‐2000 Nitrogen Analyzer (LECO Corporation, Model No. 602‐00‐500), and percent dry matter (DM) and ash were also determined (AOAC, 1995). Crude fat was determined via hexane extraction and gravimetrics (AOAC Official Method 2003.06). The amino acid contents of the samples were determined by acid hydrolysis using AOAC Official Method 982.30 (AOAC, 1995). Methionine and cysteine were determined by the performic acid oxidized hydrolysis procedure (AOAC Official Method 994.12), and tryptophan was determined using alkaline hydrolysis (ISO, 2005; Nosworthy, Franczyk, et al., 2017).

### 2.4 Protein digestibility‐corrected amino acid score

Diets were formulated, in accordance with the regulatory evaluation of PDCAAS, to contain 10% protein, supplied by the hemp ingredient, 10% total fat (total of residual hemp fat and supplemental corn oil), and 5% cellulose, with the remaining energy derived from corn starch. The hemp heart ingredient had to undergo a defatting process in order for the diet to achieve a final dietary fat content of 10%. Vitamins and minerals (AIN‐93 formulations; Harlan Teklad) were added to diets to meet the micronutrient requirements of laboratory rats. Male weanling Sprague–Dawley laboratory rats (*n* = 40 (10 per treatment); initial weight 70 g) were procured from Charles River Laboratories, randomly assigned to one of 4 diets and individually housed in suspended wire‐bottomed cages, with absorbent paper placed underneath, and treated as previously described (Nosworthy & House, 2017). Briefly, water was provided to permit ad libitum consumption, while feed was restricted to a maximum of 15 g/day over a four‐day acclimation period followed by a five‐day balance period. Feed intake was recorded during this time. During the balance period, all fecal pellets were collected, followed by air‐drying and analysis for both DM and nitrogen content. True protein digestibility (TPD%) was calculated as follows, where nitrogen intake and fecal nitrogen loss represent the product of food intake or fecal weights and their respective nitrogen values:

$$\begin{array}{c} \mathrm{TPD}\%=\left(\left(\text{Nitrogen intake}–\left(\text{Fecal nitrogen loss}–\text{Metabolic nitrogen loss}\right)\right)\right.\\ \;\left./\text{Nitrogen intake}\right)\;x\;100 \end{array}$$

The value for metabolic nitrogen loss was determined as the amount of fecal nitrogen produced by rats consuming a protein‐free diet. The PDCAAS was calculated as the product of the amino acid score and the TPD%. The amino acid score was determined by generating the ratio between the amino acid composition of the diet ingredient and the amino acid requirement pattern of a 2–5‐year‐old child, as set by the FAO/WHO, and selecting the lowest ratio generated (World Health Organization, 1991).

### 2.5 Digestible indispensable amino acid score (DIAAS)

DIAAS was calculated using the amino acid reference pattern for children aged 6 months to 3 years (World Health Organization, 2011) in conjunction with the following equation:

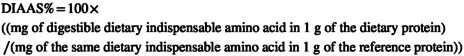




Although it is suggested that ileal amino acid digestibility be used for the calculation of DIAAS, this study only employed the true fecal nitrogen digestibility values. At the time of this publication, the use of fecal digestibility is considered acceptable until such time as a dataset of true ileal digestibility is developed (World Health Organization, 2011).

### 2.6 In vitro protein digestibility‐corrected amino acid score

An in vitro protein digestibility (IVPD) assay was also performed on each sample provided (Hsu et al., 1977; Nosworthy, Neufeld, et al., 2017; Tinus et al., 2012). Briefly, 62.5 mg of protein was heated to 37°C, adjusted to pH 8.00 ± 0.05, and the stability of that solution monitored for 10 min. If the solution held a stable pH, a cocktail of trypsin, chymotrypsin, and protease was added, and the subsequent ΔpH recorded for a period of 10 min. The IVPD was calculated as follows, where the ΔpH_10 min_ is the change in pH in 10 min from the initial pH of 8.0 ± 0.05.

$$\text{IVDP}\%=65.66+18.10∙∆{\mathrm{pH}}_{10\;\min}$$

The IVPDCAAS was calculated as a product of the amino acid score and IVPD%.

### 2.7 Protein efficiency ratio

PER values were determined over a 28‐day growth period for rats consuming feed ad libitum (HealthCanada, 1981). The animals used in the determination of PDCAAS were also used in the evaluation of PER; therefore, the 28‐day growth period included the 9‐day protein digestibility study period. Rat weights were recorded throughout the acclimation and balance periods, and feed intake was recorded throughout the study. The PER was calculated as follows:

$$\mathrm{PER}=\text{Amount of weight gain}\;\left(g\right)/\text{Amount}\;\left(g\right)\;\text{of protein consumed}.$$

Values were also adjusted to a standardized 2.5 PER value for the casein reference.

### 2.8 Statistics

Unadjusted PER values were compared via one‐way ANOVA with post‐hoc analysis using Tukey's multiple comparison test, while the relationship between in vivo and in vitro digestibilities and corrected amino acid scores was determined via regression analysis (GraphPad Prism, 7.0).

## 3 RESULTS AND DISCUSSION

### 3.1 Proximate analysis

The DM, protein, and fat content of the hemp samples are presented in Table 1. The DM of the three samples was similar to those previously reported for hemp seed and hemp protein isolates (House et al., 2010;Tang et al., 2006; Wang et al., 2008). It is important to note that while the defatted hemp hearts had the lowest fat content (0.89%), compared with hemp protein 1 (9.44%) and hemp protein 2 (6.68%), the fat content of the unprocessed hemp hearts was 47.71%. Previous work has found that the fat content of hemp hearts is approximately 47% (House et al., 2010), which is similar to the value for unprocessed hemp hearts in this study. Hemp protein isolates, which are generated via wet extraction conditions, typically contain less than 1% fat (Wang et al., 2008), which indicates that the processing method used to create the protein isolates also removes significantly more fat than that used to generate hemp proteins 1 and 2. The protein content of the defatted hemp hearts was 75.68%, which, when determined on an as‐is basis, is 36.11%, which is comparable to previous work (House et al., 2010). The protein content of hemp protein 2, 53.56%, and hemp protein 1, 66.23%, is lower than that of hemp protein isolates, 87–93% (Tang et al., 2006; Wang et al., 2008), due to the procedures used to generate the final products.

**TABLE 1:** Proximate analysis and amino acid composition of casein and hemp ingredients.

	% DM	% CP^a^	%CF^b^	ASP	THR	SER	GLU	PRO	GLY	ALA	CYS	VAL	MET	ILE	LEU	TYR	PHE	HIS	LYS	ARG	TRP
Casein	92.36	91.46	0.02	5.46	3.23	4.14	17.66	8.36	1.27	2.17	0.26	5.05	2.38	4.17	7.25	4.66	4.15	2.32	6.37	2.99	1.32
Defatted hemp hearts	95.92	75.68	0.98	6.11	1.92	2.49	10.42	2.07	2.19	2.23	1.02	2.94	1.44	2.43	3.84	2.28	2.80	1.70	2.14	8.19	0.97
Hemp protein 1	95.97	66.23	9.44	5.10	1.65	2.08	8.19	1.70	1.84	1.89	0.79	2.52	1.20	2.05	3.23	1.86	2.42	1.37	1.77	6.61	0.80
Hemp protein 2	93.02	53.56	6.68	4.30	1.39	1.94	7.40	1.43	1.61	1.61	0.73	2.05	1.03	1.67	2.72	1.51	1.95	1.16	1.57	5.48	0.69

Abbreviation: ALA, Alanine; ARG, Arginine; ASP, Aspartate; CYS, Cystine; DM, dry matter content; GLU, Glutamate; GLY, Glycine; HIS, Histidine; ILE, Isoleucine; LEU, Leucine; LYS, Lysine; MET, Methionine; PHE, Phenylalanine; PRO, Proline; SER, Serine; THR, Threonine; TRP, Tryptophan; TYR, Tyrosine; VAL, Valine.

^a^ CP = crude protein = nitrogen content (determined by LECO analysis) × 6.25.

^b^ CF = crude fat determined by hexane extraction. Amino acids are presented on a % as‐is basis.

### 3.2 Amino acid score and protein digestibility

The amino acid composition is presented in Table 1, with the resulting amino acid scores presented in Table 2. The first limiting amino acid for all hemp samples in this study was lysine, similar to previous work (House et al., 2010). The amino acid scores ranged from 0.46 for hemp protein 1 to 0.50 for hemp protein 2. Previously determined amino acid scores for hemp hearts were 0.61, while the lysine amino acid scores of protein isolates ranged from 0.57 to 0.72 (Wang et al., 2008). A study investigating the amino acid composition of dioecious and monoecious varieties of hemp determined amino acid scores between 0.69 and 0.79 (Russo & Reggiani, 2015). The difference between the amino acid scores across studies is most likely due to the use of different varieties of hemp and, in the case of enriched protein fractions, preparatory methods.

**TABLE 2:** Amino acid scores of casein and hemp ingredients.

	THR	VAL	M + C	ILE	LEU	P + T	HIS	LYS	TRP
Casein	**1.04**	1.58	1.15	1.63	1.20	1.53	1.34	1.20	1.31
Defatted Hemp Hearts	0.75	1.11	1.30	1.15	0.77	1.06	1.18	**0.49**	1.17
Hemp Protein 1	0.73	1.08	1.20	1.11	0.74	1.03	1.09	**0.46**	1.10
Hemp Protein 2	0.77	1.09	1.31	1.12	0.77	1.02	1.14	**0.50**	1.17

*Note*: Bolded values indicate the first limiting amino acid.

Abbreviations: HIS, Histidine; ILE, Isoleucine; LEU, Leucine; LYS, Lysine; M + C, methionine + cysteine; P + T, phenylalanine + tyrosine; THR, Threonine; TRP, Trypsophan; VAL, Valine.

The protein digestibility of the hemp samples was determined using both in vivo and in vitro techniques, and the values are presented in Table 3. The in vivo digestibility of hemp protein 2, 87.01%, was significantly lower than that of defatted hemp hearts, 90.39%, or hemp protein 1, 91.88% (*p* < .05). These values are comparable to the range of 85–95% found in another rodent study (House et al., 2010). It is worth noting that the use of fecal digestibility can overestimate the digestibility and therefore the overall nutritional value of a protein source. The in vitro protein digestibility ranged from 75% for defatted hemp hearts to 82%, for hemp protein 1. A different study found the in vitro digestibility of hemp protein isolates to be between 88 and 91%; however, the methodology used in that study incorporated different digestive enzymes and a longer digestion time than that used in the current study (Wang et al., 2008). Furthermore, the regression used in the current study estimates protein digestibility on an apparent protein digestibility basis, which generally produces lower values than TPD. The relationship between in vitro and in vitro determination of protein digestibility was also determined through correlational analysis; however, there was no significant correlation between these measurements (*R* ^2^ = .293, *p* = .459).

**TABLE 3:** Adjusted protein efficiency ratio, protein digestibility‐corrected amino acid scores, and the in vitro protein digestibility‐corrected amino acid scores of casein and hemp‐based protein ingredients.

	Adj. PER	AAS	%TPD	PDCAAS	IVPD	IVPDCAAS
Casein	2.5	1.04	96.31 (1.79)^A^	100	89.28 (0.13)	92.85
Defatted hemp hearts	2.14	0.49	90.39 (1.71)^B^	44.02	74.71 (0.77)	36.61
Hemp protein 1	1.75	0.46	91.88 (0.86)^B^	42.31	81.50 (0.13)	37.49
Hemp protein 2	1.94	0.5	87.01 (2.38)^C^	43.89	75.53 (0.13)	37.77

*Note*: Numbers in parentheses indicate SD where applicable. TPD was analyzed via one‐way ANOVA with Tukey's post‐hoc test. Means followed by different letters indicate a significant difference between samples (*p* < .05).

Abbreviations: AAS, amino acid score; %TPD, % true protein digestibility; PDCAAS, protein digestibility corrected amino acid score; IVPD, in vitro protein digestibility; IVPDCAAS, in vitro protein digestibility corrected amino acid score. PDCAAS is calculated as the product of AAS and %TPD, while IVPDCAAS is the product of AAS and IVPD.

### 3.3 Measurements of protein quality

The values for PDCAAS and in vitro PDCAAS are presented in Table 3. The PDCAAS of hemp products in this study ranged from 42.3%, for hemp protein 1, to 44%, for defatted hemp hearts. These values are lower than previously reported PDCAAS for either hemp seed, 51%, hemp hearts, 61%, or hemp seed meal, 48% (House et al., 2010). This discrepancy is due to the differences in amino acid scores between studies, which are dependent on the samples investigated. A PDCAAS score of approximately 43% for hemp products is similar to that of protein concentrates derived from rice and faba beans (Hughes et al., 2011; Rutherfurd et al., 2015). In all cases, the in vitro PDCAAS values were lower than their in vivo counterparts by between approximately 10%–16% due to the lower protein digestibility determined by the in vitro assay. Interestingly, the defatted hemp hearts went from the highest PDCAAS value of 44.02 to the lowest IVPDCAAS value of 36.61. This may be due to a matrix effect found in the defatted hemp hearts that was overcome in the animal model but not in the in vitro pH drop used in this study. A study on protein‐enriched hemp fractions determined amino acid scores and protein digestibility, and using those values, with a median digestibility of 89.5%, in vitro PDCAAS scores ranged from 51% to 64% (Wang et al., 2008). It is important to note that the in vitro digestibility calculated by Wang et al. 2008 was determined using a different methodology than that presented here, which would account for the higher protein digestibility and the resulting higher in vitro PDCAAS. Similar to protein digestibility, a correlational analysis was also performed between in vitro PDCAAS and PDCAAS. In this case, the correlation was significant (*R* ^2^ = .989, *p* = .005), indicating that once the amino acid score is applied to protein digestibility, the relationship between in vivo and in vitro analyses of PDCAAS are related. This relationship has also been demonstrated in other plant protein sources with correlations of *R* ^2^ = 0.9898, 0.9280, and 0.9442 (Nosworthy, Franczyk, et al., 2017; Nosworthy & House, 2017; Tavano et al., 2016).

The values for DIAAS are presented in Table 4. These values were calculated using fecal protein digestibility as suggested by the FAO/WHO as a temporary measure until sufficient ileal amino acid digestibility values become available (World Health Organization, 2011). The use of individual amino acid digestibilities rather than fecal nitrogen digestibilities would remove the concern regarding overestimation of protein digestibility described above. The DIAAS was greater for all hemp products and casein when compared with the determined PDCAAS. The greater DIAAS values were due to a decrease in the suggested requirement for lysine in the DIAAS method, 57 mg/g protein, compared with PDCAAS, 58 mg/g protein, resulting in an increased amino acid score for hemp products (World Health Organization, 1991, 2011). The value for casein via the DIAAS method, 1.03, is greater than that of PDCAAS, 1.0 (100%), as PDCAAS requires truncation of the value to 1, while that requirement is not present in the DIAAS method (World Health Organization, 1991, 2011). While there is no other data on hemp DIAAS available, scores of approximately 0.45 place these products in the same area as wheat (0.45), wheat bran (0.41), and roasted peanuts (0.43), and greater than that of rice protein concentrate (0.37) (Mathai et al., 2017; Rutherfurd et al., 2015). It is worthwhile to note that these DIAAS values are less than 0.75, the threshold for protein source claims based on FAO/WHO recommendations.

**TABLE 4:** Digestible indispensable amino acid scores of casein and hemp‐based protein ingredients.

	THR	VAL	M + C	ILE	LEU	P + T	HIS	LYS	TRP	DIAAS
Casein	1.1	1.24	**1.03**	1.37	1.16	1.79	1.22	1.18	1.64	1.03
Defatted hemp hearts	0.74	0.82	1.09	0.91	0.7	1.17	1.01	**0.45**	1.36	0.45
Hemp protein 1	0.74	0.81	1.02	0.89	0.68	1.14	0.95	**0.43**	1.31	0.43
Hemp protein 2	0.73	0.77	1.06	0.85	0.67	1.08	0.94	**0.45**	1.32	0.45

*Note*: DIAAS was calculated using true protein digestibility. Bolded values reflect the first limiting amino acid. DIAAS = digestible indispensable amino acid score.

Abbreviations: M + C, methionine + cysteine; P + T, phenylalanine + tyrosine.

**FIGURE 1:**
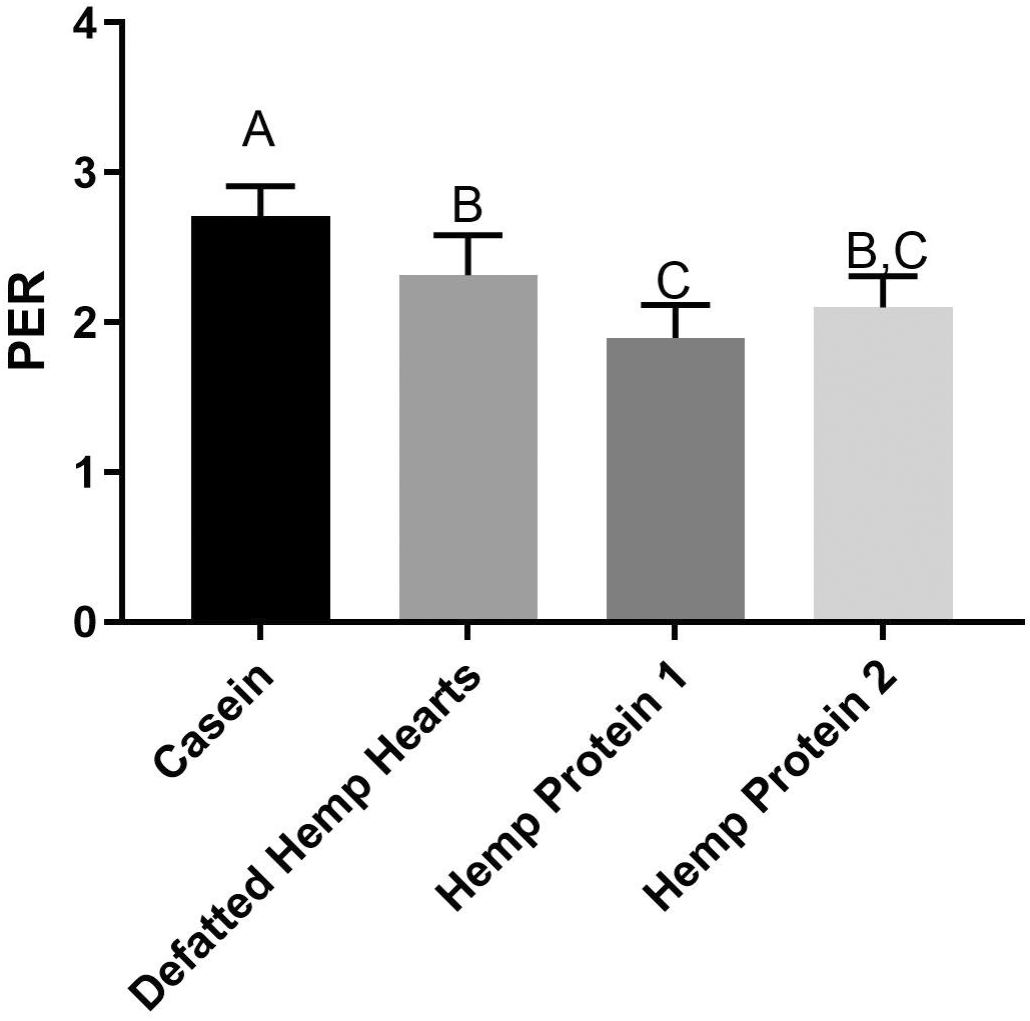
Protein efficiency ratio (PER) values for casein and hemp based protein ingredients. Mean ± SD (*n* = 10). Data were analyzed via one‐way ANOVA with Tukey's post‐hoc test. Significant differences between ingredients are denoted by different capital letters.

The PER values are presented in Figure 1. The PER of hemp protein 1, 1.9, was significantly lower than that of defatted hemp hearts, 2.3 (*p* < 0.05), while the PER of casein, 2.7, was significantly higher than any hemp product. Another study determined the PER values of hemp to be between 1.6 and 1.9, generally lower than the values determined for the hemp products investigated in this study (House et al., 2010). As a method of standardization, PER values undergo adjustment relative to the PER of casein; this normalization of PER to a casein value of 2.5 accounts for inter‐laboratory and inter‐run variation. The adjusted PER values are presented in Table 3. The adjusted PER values for hemp products investigated in this study are higher than those of cooked beans, lentils, and peas, as well as oatmeal and rice (Marinangeli & House, 2017; Nosworthy, Franczyk, et al., 2017). This positions hemp hearts and enriched hemp fractions as reliable sources of protein for growth.

### 3.4 Summary

This study assessed the protein quality of defatted hemp hearts as well as the protein‐enriched hemp products using in vivo and in vitro methods. The limiting amino acid score for all hemp products was lysine, and PDCAAS scores were similar for all three products. While there was no relationship between the in vivo and in vitro measurements of protein digestibility, there was a significant correlation between PDCAAS determined in vivo and in vitro PDCAAS. The potential for hemp products to stimulate growth was also assessed via the PER, where it was determined that while hemp protein 1 had a lower PER than defatted hemp hearts or hemp protein 2, all hemp products had PER values comparable to, or higher than, other plant‐based proteins. These data highlight the nutritional capacity of hemp protein sources while also demonstrating the relationship between in vivo and in vitro methods for determining protein quality.

## AUTHOR CONTRIBUTIONS

**Matthew G. Nosworthy:** Formal analysis (lead); investigation (lead); supervision (equal); writing – original draft (lead); writing – review and editing (lead). **Adam J Franczyk:** Formal analysis (supporting); investigation (supporting); writing – review and editing (supporting). **Jason Neufeld:** Formal analysis (supporting); investigation (supporting); methodology (supporting); writing – review and editing (supporting). **James D. House:** Conceptualization (lead); funding acquisition (lead); methodology (lead); project administration (lead); resources (lead); supervision (equal); writing – review and editing (supporting).

## FUNDING INFORMATION

Funding for this work was provided through a technical services agreement with the University of Manitoba.

## CONFLICT OF INTEREST STATEMENT

The authors declare no conflicts of interests.

## ETHICS STATEMENT

All procedures were approved by the Institutional Animal Care Committee in accordance with the guidelines of the Canadian Council on Animal Care (Protocol Number F2012‐035).

## Data Availability

The data that support the findings of this study are available from the corresponding author upon reasonable request.
